# Evaluation of Biodegradabilities of Biosynthetic Polyhydroxyalkanoates in Thailand Seawater and Toxicity Assessment of Environmental Safety Levels

**DOI:** 10.3390/polym14030428

**Published:** 2022-01-21

**Authors:** Nuttapol Tanadchangsaeng, Anchana Pattanasupong

**Affiliations:** 1 College of Biomedical Engineering, Rangsit University, 52/347 Phahonyothin Road, Lak-Hok, Pathumthani 12000, Thailand; 2 Material Biodegradation Testing Laboratory, Material Properties Analysis and Development Centre, Thailand Institute of Scientific and Technological Research (TISTR), 35 Technopolis, Tambon Khlong Ha, Amphoe Khlong Luang, Pathumthani 12120, Thailand; anchana@tistr.or.th

**Keywords:** biodegradation, biosynthesized polyesters, polyhydroxyalkanoates, seawater, residual toxicity assessment

## Abstract

Every year, thousands of tons of non-biodegradable plastic products are dumped into marine environments in Thailand’s territorial seawater, impacting various marine animals. Recently, there has been a surge in interest in biodegradable plastics as a solution for aquatic environments. However, in Thailand’s coastal waters, no suitable biodegradable plastic has been used as ocean-biodegradable packaging. Among them, polyhydroxyalkanoates (PHAs) have excellent biodegradability even in seawater, which is the desired property for packaging applications in tourist places such as plastic bags and bottles. In this report, we assess the environment’s safety and study the biodegradation in Thailand seawater of polyhydroxybutyrate (PHB) and PHA copolymer (PHBVV) that were successfully synthesized by bacteria with similar molecular weight. The two types of extracted PHA samples were preliminary biodegradability tested in the marine environment compared with cellulose and polyethylene. Within 28 days, PHB and PHBVV could be biodegraded in both natural and synthetic seawater with 61.2 and 96.5%, respectively. Furthermore, we assessed residual toxicity after biodegradation for environmental safety using seawater samples containing residual digested compounds and the standard guide for acute toxicity tests. It was discovered that marine water mites (*Artemia franciscana*) have 100 percent viability, indicating that they are non-toxic to the marine environment.

## 1. Introduction

According to the data of the garbage situation in Thailand, Thai people have created 1.15 kg/person/day, equivalent to 76,164 tons/day, giving rise to the amount of plastic waste contaminated into the sea up to 32,600 tons/year [1]. Most of the marine waste, up to 80%, comes from land-based activities such as communities, dumpsites on the harbor side, and beach tourism. The remaining 20 percent comes from marine activities such as sea transportation, fishery, and marine tourism. Plastic waste in the sea directly affects marine ecosystems, such as coral reefs, seagrass, and mangrove forests. The animals are killed either by choking on floating plastic items or becoming entangled in plastic debris. Moreover, it also affects tourism from deteriorated scenery and causes health problems by the contaminated microplastics from which plastics can be broken down into smaller sizes by sunlight (photodegradation). These give rise to some toxic chemicals dissolving into the seawater, while some plastics can still absorb toxic substances in seawater into the food chain. When aquatic animals eat, they can pass it on to the consumers in a higher order. As a result of this problem, biodegradable or compostable bioplastics are an important alternative to Thailand’s marine ecosystem development. Nowadays, many kinds of bioplastics are extensively studied and employed, such as polyhydroxyalkanoates (PHAs) [2], which we investigated in this research. Polyhydroxybutyrate (PHB) is a polymer short-chain aliphatic polyester belonging to the group of PHAs and can be produced by accumulating carbon compounds in microbial cells that synthesize the biopolyesters such as *Ralstonia euthopha* [3,4]. Under some situations, such as a scarcity of nitrogen, phosphorus, sulfur, potassium, or oxygen, the bacteria produce more PHB [5]. Besides, PHB can also be produced by microalgae (cyanobacteria) [6] and transgenic plants [7]. PHB has received much attention in the plastic industry due to the chemical and physical properties that are similar to those of synthetic polymers, especially polypropylene [8,9]. It can be used to generate various products in the form of packaging and medical equipment [10,11]. In the initial research, glucose, fructose, and sucrose are used as a source of carbon for microbes to produce PHB, which has a higher production cost than the production of polymers from petrochemicals [12]. Currently, there has been developed technology using carbon sources from waste-stream glycerol or co-substrates of glycerol and levulinic acid (LA), which are inexpensive for PHA production by bacteria [13]. Furthermore, microorganisms can use LA to create PHA terpolyester consisting of 3-hydroxybutyrate (3HB), 3-hydroxyvalerate (3HV) and 4-hydroxyvalerate (4HV) monomers that are P(3HB-*co*-3HV-*co*-4HV) or PHBVV [14].

This report evaluated the preliminary biodegradation and toxicity of PHB and PHBVV bioplastics in Thailand seawater samples using different methods to prepare both types of polymer samples in similar molecular sizes. Previous studies measured weight loss, oxygen consumption as biological oxygen demand (BOD), or CO_2_ evolution to assess the biodegradability of possibly biodegradable polymers in seawater [15,16]. The samples in this study were tested in natural and synthetic seawater modified from standard methods by analyzing evolved carbon dioxide from biodegradable activity in seawater and determining aerobic biodegradation of plastic materials in the marine environment. Additionally, an initial assessment of residual toxicity levels of the seawater after PHAs biodegradation test using the marine crustacean *Artemia franciscana* was also conducted to investigate the possibility of using PHAs as packaging items and marine equipment that do not become harmful to the marine and coastal environments.

## 2. Materials and Methods

### 2.1. Biosynthesis of Microbial PHB and PHBVV Polyesters

A laboratory mutant strain of *Ralstonia eutropha* (ATCC 17699) was used in this study. PHB and PHBVV were produced in a glycerol-rich and/or levulinic acid (LA) environment, respectively. Initial culture media for PHB and PHBVV synthesis is a mineral solution (MS) that combines glycerol 20 g/L, NaH_2_PO_4_ 1.2 g/L, (NH4)_2_SO_4_ 2 g/L, MgSO_4_·7H_2_O 0.5 g/L, K_2_HPO_4_ 7.34 g/L, and trace element 1 mL/l (consisting of 700 μM Fe(NH_4_)SO_4_, 17 μM ZnSO_4_·7H_2_O, 25 μM MnCl_2_·4H_2_O, 8 μM CuSO_4_·5H_2_O, 7.2 μM NaB_4_O_2_·10H_2_O, and 8.3 μM NaMoO_4_·2H_2_O). The *Ralstonia eutropha* was stimulated to produce PHB and PHBVV polyesters by enrichment techniques in subculture five times to find an adaptable PHA-producing cell. Pre-cultivation of *Ralstonia eutropha* cells was performed in 5 mL nutrient broth and 50% glucose 0.05 mL with 50% glycerol 0.05 mL for 24 h. Then the cells were transferred into 50 mL MS containing 20 g/L of glycerol and incubate at 30 °C for 48 h with a 200-rpm rotary incubating shaker.

A 200 mL seed flask culture was inoculated into a bioreactor. Fermentation is carried out in a 5 L Bioreactor (MDFT-N-5L/B.E.Marubishi/Thailand) containing the 2.5 L mineral solution described above, with a culture period of 62 hours. The temperature, pH, and dissolved oxygen probes monitored and controlled the fermentation. The initial OD of the wavelength of 620 nm is approximately 1.4–1.6 with culture conditions at a temperature of 30 °C, pH 6.8, which is controlled by the pH-stat system with a pH and DO controller (Mettler Toledo/Switzerland). Agitation speeds were 200 rpm or above to control the dissolved oxygen at 10% of air saturation.

For PHB biosynthesis as described previously [17], the fermentation is continuous beginning with 2.5 L of MS medium and 20 g/L of glycerol at a C/N ratio of 5. After the cell dry weight reaches the desired level (approximately 5 g/L), a glycerol solution is added using a peristaltic pump at a speed of 0.1 mL/min to allow the system to enter the growth phase. The initial pH is controlled by adding 15% ammonia solution. When the cell dry weight reaches the desired state (the value of 80 at OD_600_), it is adjusted to the PHA accumulation phase, which the pH control from ammonia will be replaced by a 5N NaOH solution. Then, the feed rate of the glycerol carbon solution was adjusted to 0.2 mL/min to start the nitrogen limiting and PHB accumulation conditions.

For PHBVV biosynthesis as previously described [14], the fed-batch fermentation began with 2.5 L of the mineral solution containing 20 g/L of glycerol and an initial C/N ratio of 5. After the cell dry weight reached the desired level (5 g/L), a 750 g/L glycerol solution was added via a pump to keep the residual glycerol concentration above 10 g/L. To maintain a C/N ratio of about 5, a solution of 15% ammonia water was added to the culture for initial pH control and nitrogen feeding. When the cell dry weight reached the desired level (80 at OD_600_) and no additional nitrogen was required, the ammonia nitrogen solution and the glycerol solution were replaced with a NaOH/KOH (5M/5M) solution and a glycerol:levulinate solution (7:3 ratio) to initiate the nitrogen limitation condition for PHBVV copolymer accumulation.

Aliquots of 10–15 mL culture medium were collected for analysis of OD_600_ and cell dry weight. The fermentation was stopped when the OD_600_ started to decrease. At 62 h of cultivation, the cells were harvested, centrifuged, lyophilized, and used to determine the dry cell weight and PHA content.

### 2.2. Extraction and Characterization of PHAs

For PHBVV extraction, the polymer that accumulated in cells was extracted with chloroform (10 mL chloroform/1 g dry cells) for at least 48 h at 60 °C and purified by two rounds of precipitation with cold methanol. The extracted PHBVV sample was further aged in a vacuum at 50 °C for at least one week to reach equilibrium crystallinity prior to further analysis. Whereas, for PHB purification, the accumulated PHB polymer in bacteria was extracted by selective dissolution of cell mass as previously reported [18]. A predetermined amount of PHB-containing cell slurry was acidified with a sulfuric acid solution (2M) and sealed in glass tubes for 1–2 h, and the solution pH was adjusted to 10 with 5N NaOH. After centrifugation at 5000 g for 15 min, the pellets were washed with an equal volume of water. The washed pellets were then re-suspended in a bleaching solution for 1–2 h. White wet PHB pellets were collected by centrifugation at 5000 g for 20 minutes, rinsed with an equal volume of water, and oven dried.

For gas chromatography quantification of PHAs, 50 mg of lyophilized cells were mixed with 2 mL chloroform and 2 mL of 3 percent H_2_SO_4_ (*v*/*v*) in methanol, supplemented with 10 mg/mL benzoic acid as the internal standard, and then heated at 100 °C for 4 hours with mixing every 30 minutes. After that, the tube containing the mixture was placed at room temperature and allowed to cool overnight. The tube was then filled with 2 mL of distilled water, vortexed vigorously for 4 minutes, and left to stand overnight. The chloroform layer was used to determine PHA in milligrams by comparing it to the standard curve obtained from gas chromatography data analysis, which was performed on a gas chromatography instrument (Agilent technologies 7890A). In addition, the percentage of PHA content per dry cell weight (% *w*/*w*) was calculated as previously described [19].

To measure the molecular weight of PHAs, gel permeation chromatography (GPC) analyses were carried out on Waters 2414 refractive index (RI) detector, equipped with Styragel HR5E 7.8 × 300 mm column (molecular weight resolving range = 2000 – 4,000,000). Samples dissolved in hot chloroform were eluted with chloroform at a flow rate of 1.0 mL/min at 40 °C and calibrated with polystyrene standards.

The PHA polymer was further characterized by using nuclear magnetic resonance (NMR). PHA samples were dissolved in CDCl_3_ (25 mg/mL) via gentle mixing and heating, and analyzed with ^1^H spectroscopy (Varian Unity Inova 500 MHz) to find the monomer composition and chemical structure information.

### 2.3. Preliminary Biodegradation Test

The preliminary biodegradation method is performed for biodegradability screening of materials compared with reference material by measurement carbon dioxide (CO_2_) accumulation from the microbial degradation activities in both natural and synthetic seawater with sediment under laboratory scale test. The result is designed to percentage of biodegradation that calculated from conversion of carbon in the reference or test materials to amount of cumulative carbon dioxide. As a result, this method was carried out using modified versions of ISO 19679:2016 [20], which specified a test method for determining the degree and rate of aerobic biodegradation of plastic materials when settled on sandy marine sediment, and ASTM D6691-17 [21], which was used to measure the degree and rate of aerobic biodegradation of plastic materials exposed to a pre-grown population of at least ten aerobic marine microorganisms of recognized genera or the indigenous population found in natural seawater.

**Figure 1:**
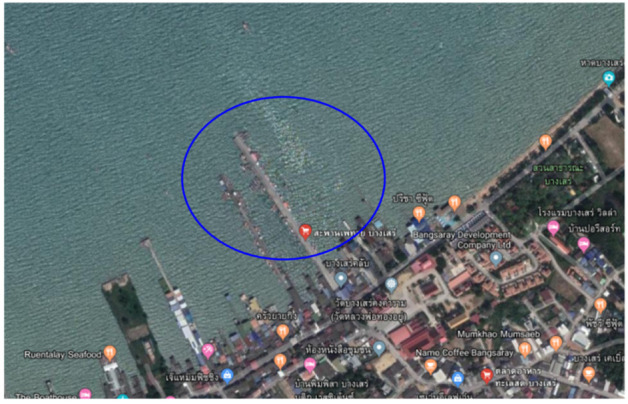
Map showing the coordinate of the seawater sampling point at Phethai bridge, Bang-Sare subdistrict, Sattahip district, Chonburi province, Thailand.

The preliminary biodegradation of PHB and PHBVV bioplastic samples was estimated in both natural and synthetic seawater with sediment by measuring the amount of accumulated carbon dioxide generated by the degradation activity of the samples within a group of effective bacteria isolated from the coast of Thailand. The bacterial consortium is composed of *Bacillus marisflavi* (TISTR 2158), *Bacillus safensis* (TISTR 2163), *Bacillus subtilis* (TISTR 2173), *Bacillus methylotrophicus* (TISTR 2193), *Pseudomonas* spp., and *Enterobacter* spp. The natural seawater sample was collected at the Phethai Bridge, Bang-Sare Subdistrict, Sattahip District, Chonburi Province, Thailand as shown in Figure 1, and the characteristics of the collected natural seawater are listed in Table 1. In parallel, the reference material that is known to biodegrade (as chromatography-grade cellulose) must be included in each test run to check the activity of the bacterial consortium. Synthetic seawater was made according to ASTM D6691 (2017) standard [22] by dissolving the following substances in 1000 mL of distilled water: 2 g NH_4_Cl, 17.5 g synthetic sea salt, 2 g MgSO_4_ 7H_2_O, 0.5 g KNO_3_ and 0.1 g K_2_HPO_4_ 3H_2_O, and subsequently autoclaved for sterilization.

For the preparation of the biodegradation test, the PHA test samples, or positive or negative references as shown in Table 2, the test size not over 1 mm, were mixed in the amount of 1% *w*/*v* with natural or synthetic seawater with a total bacterial count of 5–6 log CFU per ml. The samples were incubated in a closed condition at a temperature of 30 ± 2 °C. The amount of carbon dioxide (CO_2_) that occurs in the system from the activity of microbes was measured throughout the 28 test days in 3 repetitions per sample test. The samples were tested against positive reference and negative reference samples in closed conditions under temperature control conditions of 30 ± 2 °C at the laboratory, as shown in Table 2.

The biodegradability (% Biodegradation) of PHA polyester samples was calculated by subtracting the cumulative amount of carbon dioxide of the control blank from that of the test sample and by dividing the value by the theoretical amount of carbon dioxide (ThCO_2_) of the test sample as shown in the following equation:

(1) $$\%\;\mathrm{Biodegradation}=\frac{{\mathrm{CO}}_{2\;}(\mathrm{test})\;-{\;\mathrm{CO}}_{2\;}\left(\mathrm{Control}\right)\;}{{\mathrm{ThCO}}_{2}}\;\times \;100$$

It is noted that the total organic carbon (TOC) values of the PHB and PHBVV samples are 54.26 and 53.91 g/g, respectively.

### 2.4. Acute Toxicity Tests Using Rotifers

**Figure 2:**
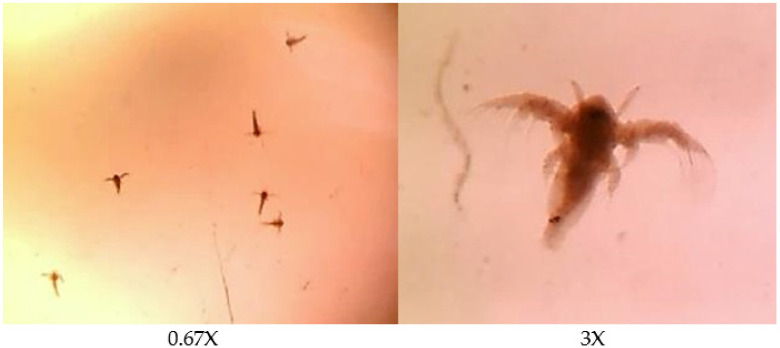
Optical micrographs of seawater mites (*Artemia franciscana*) used in this study.

The seawater solution samples of PHB and PHBVV biopolymers that have examined initial biodegradation tests in natural and synthetic seawater were subsequently assessed for the environmental safety levels by the ARTOXKIT M test set following the standard guide for acute toxicity test with the *Rotifer Brachionus* (ASTM E1440-91) by seawater mites (*Artemia franciscana*) as shown in Figure 2. The procedural details of the test are described in Snell et al. (1991), and the standardized ASTM protocol [23]. The rotifer 24-h acute toxicity test was performed as a convenient monitor for screening the most effective material according to the standard method.

### 2.5. Statistical Analysis

The SPSS Statistics program version 25.0 (IBM, Armonk, NJ, USA) and Microsoft Excel 2019 were used to analyze the data (Microsoft, Redmond, WA, USA). A paired t-test was employed to compare two sets of data. The differences in averages were judged statistically significant at the 95 percent confidence level (*p* ≤ 0.05).

## 3. Results and Discussion

### 3.1. Biosynthesis of Bacterial PHB and PHBVV Polyesters

**Figure 3:**
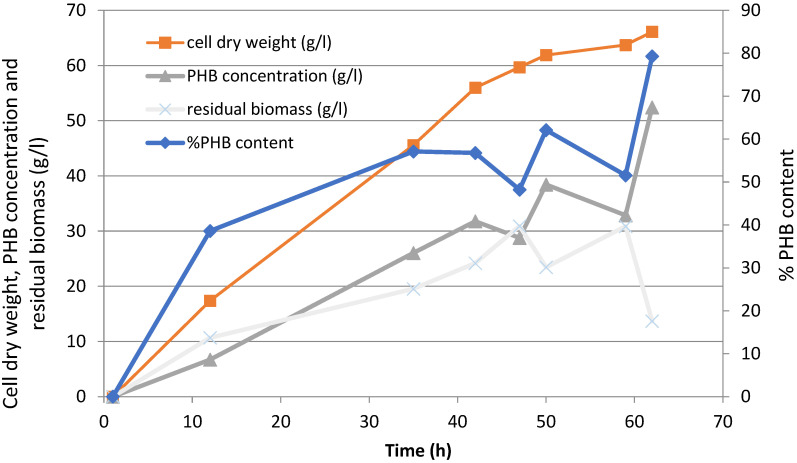
Time courses of PHB homopolyester formation: cell dry weight, PHB content, and PHA concentration cultured by fed-batch fermentation in 5 L bioreactor using glycerol as a single substrate.

The cultivation of *Ralstonia eutropha* ATCC 17,699 with glycerol as a sole carbon source in order to obtain a PHB polymer yielded the following results as shown in Figure 3. Inoculated at 2.5 L for a period of 62 h under fed-batch cultivation, the cell dry weight increased up to 68 g/L. During the feasting period of 35 h onwards, it was found that PHB was rapidly accumulated within the cells, whereas the PHB concentration was higher and residual biomass tended to be lower. PHB content increased up to 82% at the end of fermentation. Table 3 summarized the fermentation results corresponding to the PHB production rate, and the PHB productivity can be calculated at 0.8 g/L/h.

**Figure 4:**
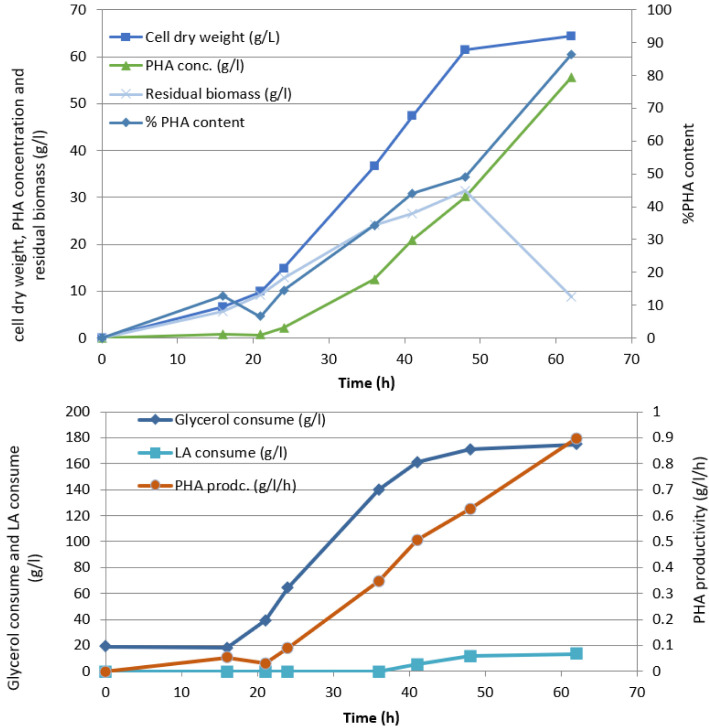
Time courses of PHBVV copolyester biosynthesis: cell dry weight, PHA content, cell-PHA productivity, and substrate consumption cultured by fed-batch fermentation in 5 L bioreactor using glycerol/levulinate co-substrates.

On the other hand, fed-batch fermentation of *Ralstonia eutropha* ATCC 17,699 was performed in a 5 L bioreactor containing 2.5 L mineral solution supplemented with a carbon source of glycerol:levulinic acid cosubstrates at a ratio of 7:3 as precursors of 3HB, 3HV and 4HV monomer unit. The cultivation of PHBVV copolyester was obtained as shown in Figure 4, providing that at the period of 20–35 and 42–62 h the cell growth rate and the PHA concentration increased dramatically to 64 g/L and 55 g/L, respectively. During the 40-h cultivation period onwards, there was a rapid accumulation of PHA within the cells giving rise to the concentration of PHA being higher and the residual biomass tended to be lower. The PHA content increased as high as 83% at the end of fermentation harvested at 62 h. We found that the rate of cell production in the bioreactor tended to increase over time relating to PHBVV biosynthesis. In addition, the graph shows that levulinic acid was applied at approximately 40 h, which is the PHA accumulation period resulting in the PHA productivity increased up to 0.9 g/L/h.

Table 3 listed PHA copolymer accumulation observed in the PHBVV terpolymer consisting of 3HB 87 mol%, 3HV 12 mol% and 4HV 1 mol%, which affect the mechanical property of PHA polymer by making it more flexible. The molecular weight and polydispersity of PHB homopolyester were at 95 kDa and 1.71 and of PHBVV biopolyester, which was at 163 kDa and 1.98 (similar to commercial PHA), appropriate for further polymer processing for packaging application [24,25].

It should be mentioned that PHB and PHBVV biosynthesis from glycerol and levulinic acid is capable of meeting the requisite yields of cell mass and PHA production with acceptable molecular size and chemical composition. Normally, bacterially synthesized PHB homopolymer has a higher amount of M_w_ than that of PHA copolymer. However, in this study, we performed different extraction and purification methods on PHA and PHBVV. The PHB sample was dissolved by dissolution of non-PHB cell mass while the PHBVV sample was extracted using the chloroform/methanol precipitation method. As a result, the values of molecular weight and PDI of both PHB and PHBVV are very similar giving rise to the suitable specimen for biodegradation tests as initial characteristics of the samples ideally should be the same.

**Figure 5:**
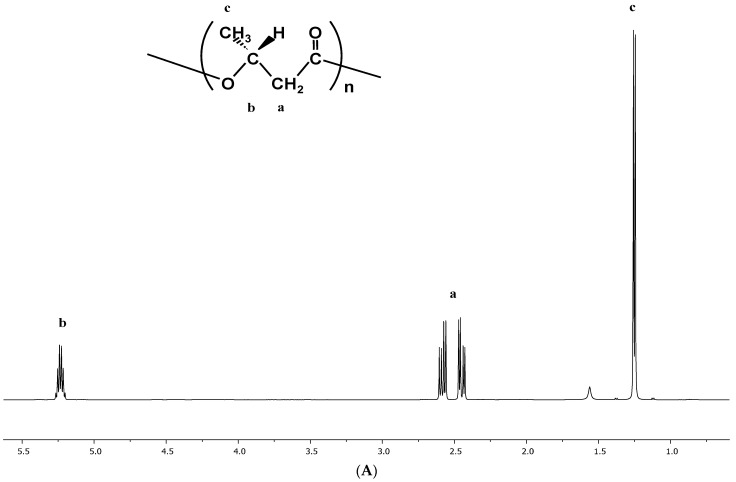

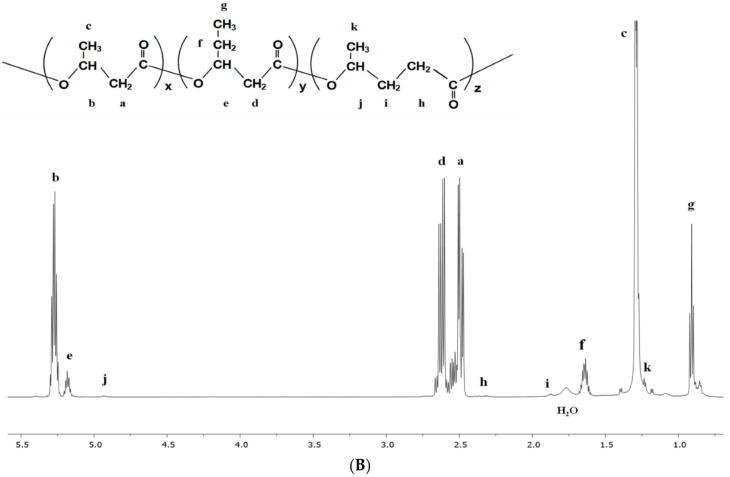
^1^H-NMR spectra of the extracted PHA samples used in biodegradation test in seawater: (**A**) PHB, (**B**) PHBVV.

The NMR measurement confirmed the purified PHB and PHBVV samples to determine the chemical structure using the proton-NMR technique. The spectra were shown in Figure 5, and the results PHB homopolymer and PHBVV copolymer have been intrinsically produced and extracted from *Ralstonia eutropha* cells.

### 3.2. Biodegradation in Seawater of PHB and PHBVV

In this Thailand seawater degradation study, we used two biosynthetic PHA samples of PHB and PHBVV, both of which had a similar amount of molecular weight approximately 160–200 kDa with closed PDI as shown in Table 3.

Table 4 shows the percentage of biodegradation generated from the cumulative amount of carbon dioxide resulting from degradation activities in natural or synthetic seawater for 28 days by the modified methods of ISO 19679:2016 and ASTM D6691-17. PHB and PHBVV samples that were tested, compared to cellulose (R+) and polyethylene (R−) references. The results show that PHBVV had a higher biodegradability than PHB in both natural (61.2%:12.8%) and synthetic (96.5%:86%) seawaters correlated to the control and reference samples. It was noted that PHB and PHBVV copolyesters can be almost completely biodegraded in synthetic seawater within 28 days, whereas PHBVV has the ability to mineralize to CO_2_ to over half the amount of biodegradation in Thailand natural seawater.

**Figure 6:**
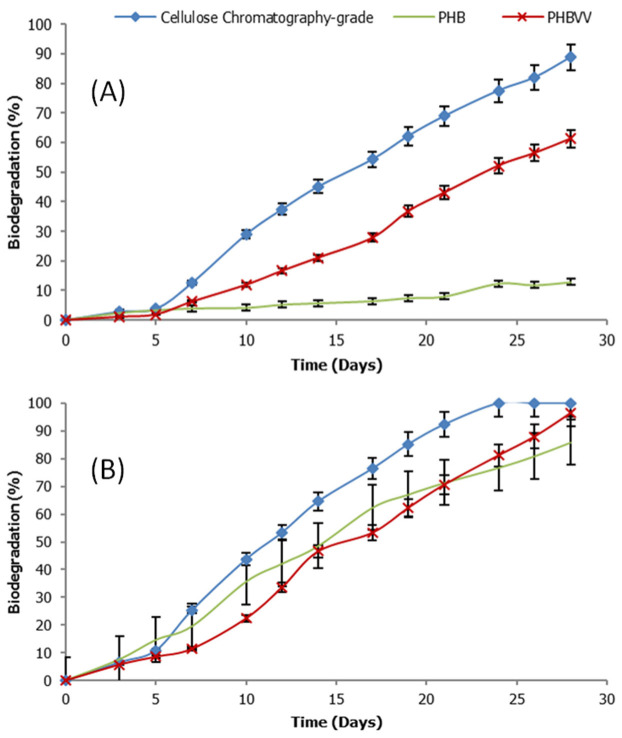
Biodegradation of PHB and PHBVV samples compared to cellulose reference sample in natural seawater (**A**) and synthetic seawater (**B**).

Figure 6 shows biodegradation curves of PHB and PHBVV samples compared to cellulose reference samples in natural seawater and synthetic seawater. In synthetic seawater, the degree of biodegradation of both the PHB and PHBVV increased with time at a rapid rate after 5 days and could be completely converted to carbon dioxide for 28 days, correlating to cellulose reference. Whereas the degree of biodegradation in Thailand’s natural seawater of PHB was almost stable with time, but that of PHBVV still increased with time up to around 60% biodegradation, suggesting that PHA microorganisms are present in natural seawater in Thailand. This indicated that PHBVV copolyester had a higher ability to be biodegraded than PHB homopolyester. Typically, copolymer composition affects a decrease of the degree of crystallinity (X_c_) of PHA copolymer giving rise to the lower PHBVV crystallinity than PHB [14]. In this case, from the enzymatic biodegradation report [12], PHA copolymer with low X_c_ has more easily biodegraded than PHB homopolymer with high X_c_, which tends to have a relationship with the biodegradation in seawater.

### 3.3. Initial Assessment of Residual Toxicity Levels of Biodegraded PHAs Seawater

The degraded PHAs seawater samples were subsequently assessed to investigate the residual toxicity of environmental safety level by using ARTOXKIT M, a 24-h assay based on the mortality of the test organisms with a calculation of the 24-h LC_50_ of *Artemia franciscana* in test solutions containing 25% and 50% of natural or synthetic seawater samples. Table 5 shows the percentage viability of *Artemia franciscana* in natural seawater samples. The survivability of all of the samples is nearly 100 percent, demonstrating that the degraded seawater of PHB and PHBVV biodegradation had no residual toxicity. Moreover, as seen in Table 6, the percentage viability of *Artemia franciscana* in synthetic seawater in the solution of ARTOXKIT M shows slightly decreased for PHBVV degraded solution, which does not impact the environmental safety level [27].

This finding indicates that Thai seawater can decompose PHA in the presence of local microorganism consortia, allowing for the usage of bioplastic PHA in coastal areas or islands. Moreover, even when degraded, it does not produce microplastic. The compounds that have been digested are also non-toxic to the marine environment. In the near future, the application of PHA to manufacture bags or marine supplies is quite likely to be used in Thailand and other countries in Southeast Asia.

## 4. Conclusions

In this research, we successfully synthesized PHB and PHBVV polyesters in fed-batch fermentation of *Ralsonia eutropha* bacterium grown on glycerol-based substrates. PHB and PHBVV samples were prepared with similar molecular weight by different extraction and recovery processes. We initiated a method for measuring the quantity of carbon dioxide accumulated from breakdown processes, which was evaluated using bacteria isolated from Thailand’s coast. Biodegradation of biosynthetic PHB and PHBVV in synthetic seawater can be over 60 and 95 percent, respectively, while natural seawater in Thailand can be over 12 and 85 percent, respectively, in 28 days. Residual toxicity levels after biodegradation using seawater mites (*Artemia franciscana*) can confirm that the percent viability of PHB and PHBV degraded solutions is nearly 100 percent viability at 24 h when compared to all control groups that have no impact on the marine environment.

## Figures and Tables

**Table 1:** Physical and chemical properties of natural seawater.

Parameters	Analysis Results
1. Temperature, °C	30.9
2. Salinity, ppt	34.0
3. pH	8.13
4. Dissolved oxygen, mg/L	6.51

**Table 2:** Sample details employed for the biodegration test in natural and synthetic seawater.

No.	Sample Test	Sample Code	Sample Details
1	ControlSample	Control	Natural seawater or Synthetic seawater
2	Positive reference sample	R+	Chromatography-grade cellulose
3	Negative reference sample	R−	Polyethylene
4	Sample 1	PHB	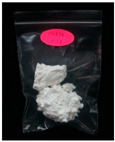
5	Sample 2	PHBVV	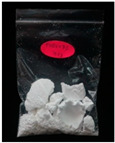

**Table 3:** Biosynthesis of harvested PHA samples after fed-batch cultivation of *Ralstonia eutropha* for 62 h.

PHA Type Sample	Cell Dry Weight (g/L)	PHA Content per Dry Cell ^a^ (%)	PHA Productivity (g/L/h)	PHA Monomer Composition ^b^			M_w_ ^c^ (kDa)	PDI ^c^
				3HB (%)	3HV (%)	4HV (%)		
PHB	68	82	0.8	100	0	0	195	1.71
PHBVV	64	83	0.9	87	12	1	163	1.98

^a^ PHA content incorporated in cells was determined by gas chromatography (GC). ^b^ PHA monomer composition was determined by 500 MHz ^1^H NMR. ^c^ Molecular weight of PHA was determined by gel permeation chromatography (GPC). (M_w_: weight average molecular weight, PDI: polydispersity index).

**Table 4:** Biodegradability of PHB and PHBVV samples in Thailand natural and synthetic seawaters compared with control, positive and negative references.

Sample Code	% Biodegradation at 28 Days			
	Natural Seawater		Synthetic Seawater	
	Average	SD	Average	SD
Control ^1/^	-	-	-	-
R+ ^2/^	88.8	6.5	100.0	0.8
R− ^3/^	0.8	1.2	5.5	8.5
PHB ^4/^	12.8	5.3	86.0	5.2
PHBVV ^4/^	61.2	3.4	96.5	7.2

Note: The percentage of biodegradation is calculated from ^1^ The amount of accumulated carbon dioxide generated by microbial activity in natural or synthetic seawater. (Not including the amount of carbon dioxide in the test container); ^2^ The cumulative amount of carbon dioxide resulting from degradation activities. Chromatography-grade cellulose by a group of microbes from the coastal sea. (Cellulose chromatography-grade easily decomposed in natural conditions: according to ASTM D6691: 2017 [21]); ^3^ The cumulative amount of carbon dioxide from polyethylene degradation activities by a group of coastal microbes. (Polyethylene is difficult to decompose in natural conditions: according to ASTM D5338-98: 2003 [26]); ^4^ The cumulative amount of carbon dioxide from the decomposition activities tested by microbial communities from the coast of Thailand.

**Table 5:** Percent viability of *Artemia franciscana* in natural seawater samples.

Percentage of Natural Seawater Samples in the Solution of ARTOXKIT M Test	% Viability at 24 h				
	Control	R+	R−	PHB	PHBVV
25%	100	100	100	100	100
50%	100	100	100	93	100

**Table 6:** Percent viability of *Artemia franciscana* in synthetic seawater.

Percentage of Synthetic Seawater Samples in the Solution of ARTOXKIT M Test	% Viability at 24 h				
	Control	R+	R−	PHB	PHBVV
25%	97	100	100	100	97
50%	67	100	97	100	87

## Data Availability

The data presented in this study are available on request from the corresponding author.
